# A Pathognomonic Sign of Acute Miliary Tuberculosis of the Lungs

**Published:** 1897-07

**Authors:** 


### A PATHOGNOMONIC SIGN OF ACUTE MILIARY TUBERCULOSIS OF THE LUNGS.

The close resemblance of certain cases of acute miliary tuberculosis to typhoid fever is remarked upon by all authorities. Even such accurate tests as Widal's and Elsner's are said to fail sometimes in the early differentiation of these two diseases. Such being the case, any diagnostic sign of real value is a welcome addition to our store of knowledge. In the *N. Y. Medical Journal* of April 17th, Dr. David Riesman again calls attention to the peculiar friction fremitus produced by the rubbing of a multitude of tubercles just beneath the pleura. The sound, he says, is heard during both inspiration and expiration. It is distinctive in character, being very soft and fine and separate from the bronchial rales. The fremitus is perceptible to the touch as a gentle rubbing, much softer than any rhonchial fremitus the writer ever observed. The case reported, a married painter twenty-five years old, lived about three weeks from the inception of the disease, and the diagnosis was confirmed by a necropsy. A similar circumstance was that during all this period of sickness the patient had no cough or cyanosis, which may be ascribed probably to deficient excitability of the medullary centres dependent on the extreme toxemia.

